# Supplementary material for: Modulation of Anionic Lipid Bilayers by Specific Interplay of Protons and Calcium Ions
Source: Biomolecules. 2022 Dec 17;12(12):1894. doi: 10.3390/biom12121894 (PMC9775051; doi:10.3390/biom12121894)
Supplement: Supplementary file 1 [file biomolecules-12-01894-s001.zip › biomolecules-1977799-supplementary.pdf]

Supplementary Information

Modulation of Anionic Lipid Bilayers by Specific Interplay of Protons and Calcium Ions

Abhinav [1], Piotr Jurkiewicz [1], Martin Hof [1], Christoph Allolio [2] and Jan Sýkora [1,*]

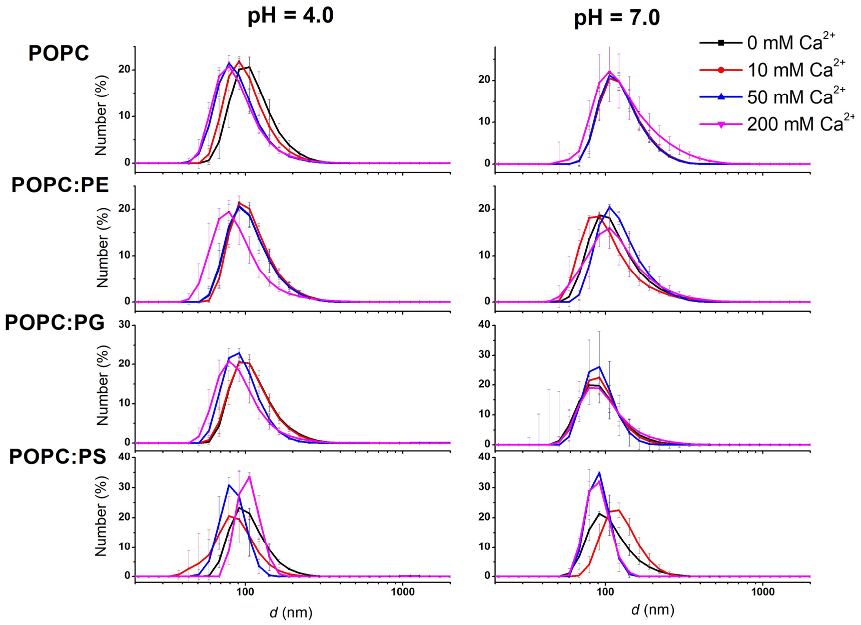

Figure S1: Number weighted size distributions of the investigated liposomes of varying compositions recorded at different Ca^{2+} concentrations. The DLS data were accumulated five times yielding 5 independent read-outs of the number weighted size distributions. These sets of 5 data sets were statistically treated yielding the standard deviation for each particular size.

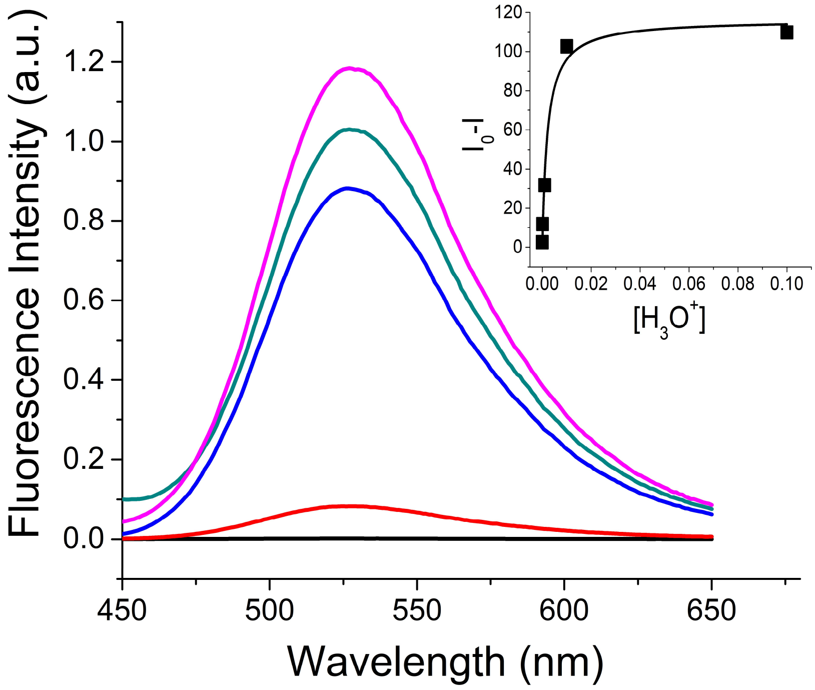

Figure S2: Emission spectra of Prodan recorded at different pHs. The fluorescence intensity gets strongly reduced with decreasing pH due to the protonation of the dimethyl-amino group of Prodan molecule. The apparent pK_a for the Prodan protonation in water was estimated to be 2.7 (gained by the hyperbolic fit of the $[H_3O^+]$ dependence on I_0-I, where I_0 and I denote the integrated emission of fully deprotonated Prodan (at pH 7.0) and at the given pH, respectively).

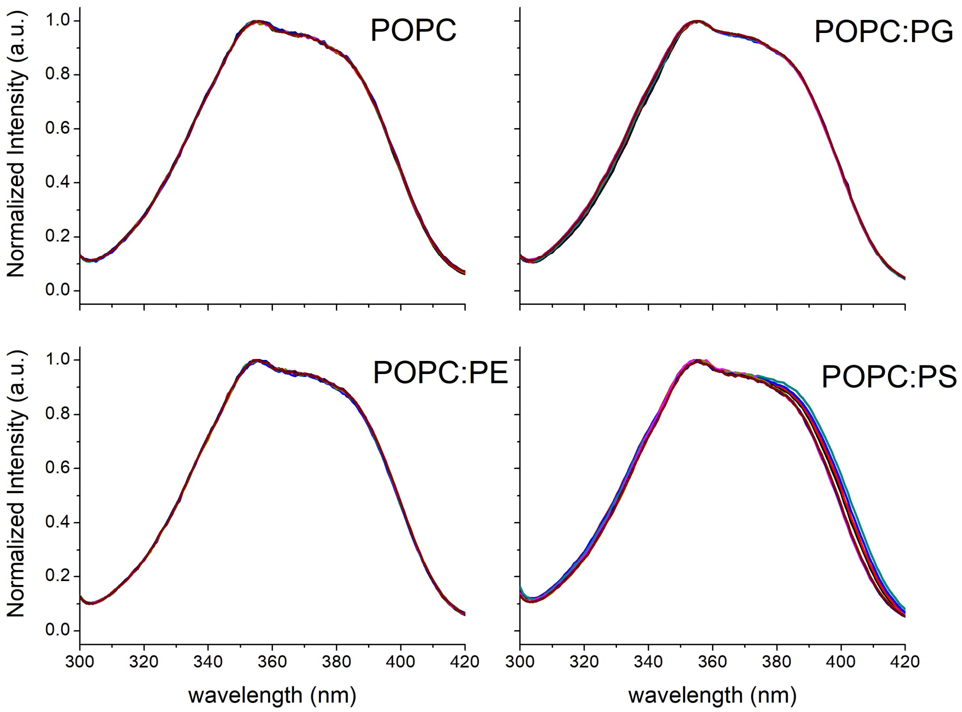

Figure S3: Excitation spectra of Laurdan embedded in the liposomes of various compositions under the investigated range of pHs and Ca^{2+} contents. No severe changes in the spectral lineshape indicate that phase separation and/or phase transition has not occurred under any of the applied conditions.
